# Skin rashes after SARS‐CoV‐2 vaccine: which relationship, if any?

**DOI:** 10.1002/iid3.428

**Published:** 2021-06-19

**Authors:** Cataldo Patruno, Maddalena Napolitano, Luca Stingeni, Gabriella Fabbrocini

**Affiliations:** ^1^ Department of Health Sciences University Magna Graecia of Catanzaro Catanzaro Italy; ^2^ Department of Medicine and Health Sciences Vincenzo Tiberio University of Molise Campobasso Italy; ^3^ Dermatology Section, Department of Medicine University of Perugia Perugia Italy; ^4^ Section of Dermatology, Department of Clinical Medicine and Surgery University of Naples Federico II Napoli Italy

**Keywords:** COVID‐19, skin reaction, vaccine

## Graphical abstract

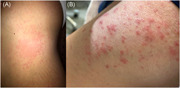

Recently, Food and Drug Administration and European Medicines Agency approved the BNT162b2 messenger RNA (mRNA) vaccine for the prevention of severe acute respiratory syndrome coronavirus 2 (SARS‐CoV‐2) infection (coronavirus disease 2019 [COVID‐19]). The vaccine is administered in two doses separated by 21 days.^1^ The Centres for Disease Control and Prevention identified the onset of anaphylaxis in 21 out of 1,893,360 subjects receiving the first vaccine dose.^2^ Furthermore, pruritic rash and/or mild respiratory symptoms were observed in 83 of them.^2^ All these adverse reactions mostly appeared within the first 30 min after the vaccination and can likely be interpreted as immunoglobulin E‐mediated hypersensitivity, hypothetically related to the vaccine component polyethylene glycol 2000.^2^, ^3^ To date, no delayed cutaneous adverse events have been reported, other than injection site inflammation.

Herein, we described two patients with skin rash appearing several days after the first doses of the vaccine. A 42‐year‐old woman with no history of allergies or skin diseases developed acute urticaria (AU) on the trunk and limbs (Figure 1A). Wheals started 7 days after vaccine administration. Systemic antihistamine treatment alone was inefficacious, while the addition of prednisone (25 mg/day) for 1 week led to healing. The second patient was a 55‐year‐old healthy man with multiple itchy erythematous papules, vesicles, and blisters on the buttocks and extensor surface of the extremities (Figure 1B). The rash appeared about 10 days after vaccination. Skin histology showed spongiosis, epidermal exocytosis of lymphocytes, and apoptotic keratinocytes, besides dermal edema. These clinical and histologic features were consistent with an erythema multiforme (EM)‐like eruption. The eruption disappeared after 10 days of treatment with systemic prednisone (25 mg/day). Both the patients denied infections or intake of drugs after the vaccine administration, thus leading to the suspicion of association between exposure to the vaccine and the appearance of the rashes. Delayed maculopapular reactions have been described after the administration of conventional vaccines for bacterial or viral diseases.^4^ These reactions may be due to both the antimicrobial or additional components of the drug.^4^ However, there are no reports regarding delayed urticarial or EM‐like eruptions.^4^ On the other hand, AU and EM‐like eruption may occur in some patients affected with COVID‐19.^5^ At least some of these cases are considered related to viral infection and not to concomitant treatments.^5^

**Figure 1:**
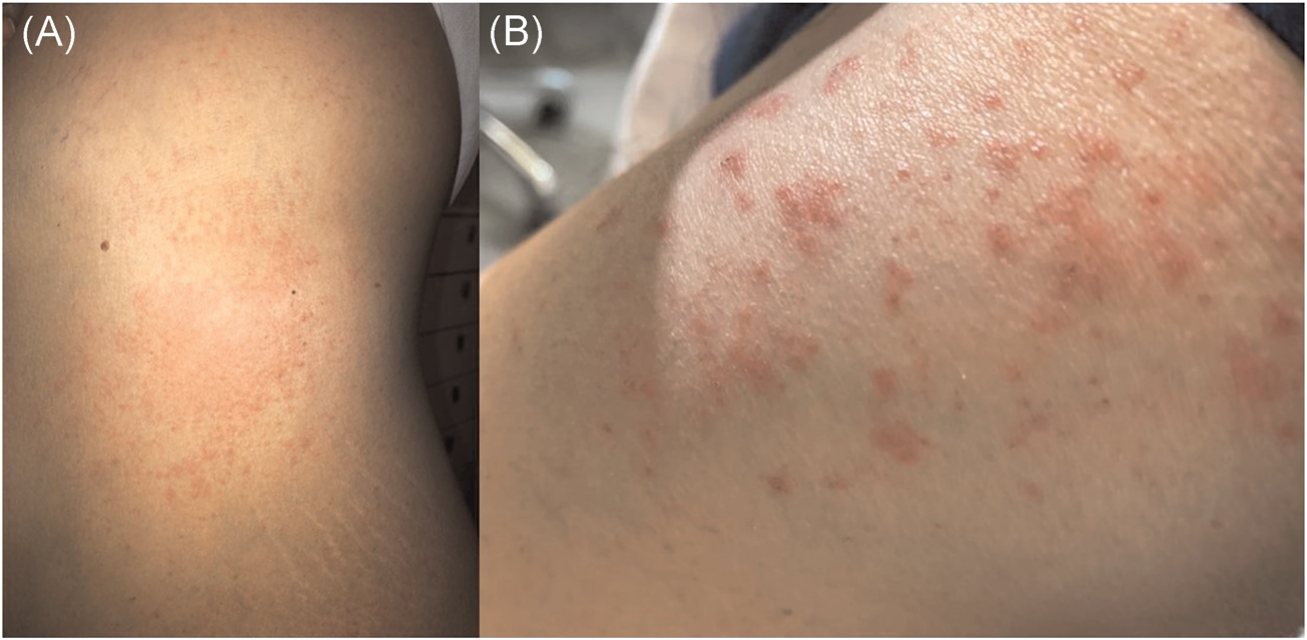
(A) Wheals on the lower limbs appeared 7 days after vaccine administration; (B) erythematous papules and vesicles of the extensor surface of the extremities appeared about 10 days after vaccination

BNT162b2 is a nucleoside‐modified mRNA vaccine that induces transient expression of the SARS‐CoV‐2 spike antigen (SA) and subsequent neutralizing antibody production and cellular response against the virus.^1^ It could be assumed that, in our two patients, the expression of SA induced by the BNT162b2 vaccine might have induced both AU and EM‐like eruptions. Therefore, the pathogenesis might be similar to that occurring in COVID‐19 patients developing these rashes. Obviously, it is essential to collect data regarding a large population to evaluate the effective association of delayed urticarial or EM‐like eruptions with the BNT162b2 vaccine. Furthermore, such cases might also be useful in evaluating the immune response and efficacy of vaccination for SARS‐CoV‐2.

## CONFLICT OF INTEREST

The authors declare they have no conflict of interests.
